# Impact of tell show do and audio-visual distraction techniques on pediatric dental anxiety: A comparative study

**DOI:** 10.6026/973206300212221

**Published:** 2025-07-31

**Authors:** Shubhranil Bhowmick, Prasanthi Bajana, Kavya S. Patel, Kanchan Sharma, R. Amirthaa Varshini, Kodali Srija, Pratik Surana

**Affiliations:** 1 Department of Pedodontics & Preventive Dentistry, Kalinga Institute of Dental Sciences, Kalinga Institute of Industrial Technology (KIIT) Deemed to be University, Bhubaneswar, Odisha, India; 2 Department of Oral Medicine and Radiology, Government Dental College and Hospital, Vijayawada, Andhra Pradesh, India; 3 Department of Pedodontics & Preventive Dentistry, Government Dental College and Hospital, Ahmedabad, Gujarat, India; 4 Department of Orthodontics and Dentofacial Orthopaedics, Awadh Dental College and Hospital, Jamshedpur, Jharkhand, India; 5 Department of Oral and Maxillofacial Surgery, Sri Ramakrishna Dental College and Hospital, Coimbatore, Tamil Nadu, India; 6 Department of Pedodontics & Preventive Dentistry, Mallareddy Dental College for Women, Mallareddy Vishwavidyapeeth (Deemed to be University) Hyderabad, India; 7 Department of Pedodontics and Preventive Dentistry, Maitri College of Dentistry and Research Centre, Durg, Chhattisgarh, India

**Keywords:** Behaviour management, tell show do, audio-visual distraction

## Abstract

Effective behavior management is crucial in pediatric dentistry to alleviate anxiety and improve treatment outcomes. The current research was conducted to compare the effectiveness of behavior management techniques, specifically Tell Show Do (TSD) and Audiovisual Distractions (AVD), on apprehension levels in children undergoing the extraction of primary teeth. Forty children aged 4 to 8 years, all experiencing their first dental visit with a tooth indicated for extraction, were assigned to Group A (TSD) and Group B (AVD). Dental anxiety was assessed using Venham's Picture Test both pre-operatively and post-operatively. Results showed a statistically significant decrease in anxiety levels for Group B, along with notable differences between the two groups. Thus, the findings suggest that the Audio-visual Distraction method is an effective approach for managing anxiety in pediatric dental patients.

## Background:

Pediatric patients frequently exhibit a wide range of responses to dental treatment, oscillating between prompt acceptance and pronounced fear or resistance. The pediatric dentist's role in addressing an anxious child extends beyond merely alleviating the presenting dental issue; it also encompasses the responsibility of educating the child on effective strategies for managing anxiety [1, 2]. The fundamental techniques of nonpharmacological interventions frequently employed in dental practice include tell-show-do, modeling, reinforcement, voice control, nonverbal communication and distraction. While numerous management techniques have proven effective in addressing anxious children, attitudes among parents and dental professionals towards aversive techniques are evolving [3]. Due to concerns over litigation, there has been a notable increase in the preference for non-aversive techniques, such as distraction, among dental professionals. These techniques aim to enhance cognitive orientation and promote coping skills for a more favorable treatment experience. "Distraction" serves as a strategy designed to redirect a patient's attention from their current behavior to engage their interest in an alternative focus, thereby assisting patients in managing brief episodes of stress [4]. Distraction techniques are frequently employed to divert a child's attention from the unpleasant aspects of on-going dental treatments [5, 6]. These techniques encompass both audio-visual and auditory methods, such as playing music and audio narratives. Evidence indicates that vibro-acoustic therapy can effectively reduce stress levels. Conversely, research has confirmed that the sounds produced by dental instruments and equipment, such as the handpiece and saliva ejector, can induce anxiety and discomfort in pediatric patients. Therefore, audio visual distraction techniques are valuable in redirecting children's focus away from the distressing sounds present in the dental environment, ultimately contributing to lessening in their anxiety levels [7]. Therefore, it is of interest to assess the effectiveness of tell show do and audiovisual distraction techniques in managing anxiety levels of children undergoing primary tooth extraction.

## Materials and Methods:

## Study design:

This study employed a randomized controlled trial design to evaluate the effectiveness of two behavior management techniques- tell show do and audio-visual distractions -in reducing dental anxiety among pediatric patients undergoing primary tooth extraction. The trial adhered to ethical guidelines; ensuring informed consent was obtained from the parents or guardians of all participating children prior to the study.

## Study subjects:

A total of 40 children, aged between 4 and 8 years, were recruited for this study. All participants were first-time dental visitors requiring primary tooth extractions due to dental caries or space maintenance issues. Children were allocated at random to one of two groups:

Group A (TSD): Participants who received the Tell Show Do technique.

Group B (AVD): Participants who received Audio-visual Distractions during the procedure.

## Inclusion criteria:

This study involves healthy children aged 4 to 8 years requiring extraction of primary teeth. They have no previous dental treatment, display Frankel behavior ratings of 3 and 4, and have no allergies to dental anesthetic agents.

## Exclusion criteria:

Children with a previous dental history, medically compromised patients, and those exhibiting Frankel behavior ratings of 1 and 2 were excluded from the study.

## Pre-operative assessment:

Before the procedure, children's baseline anxiety levels were measured using VPT, it consists of eight cards, each featuring two figures: one labeled as "anxious" and the other as "non-anxious." Children were instructed to indicate the figure they related to at that moment. The cards were presented in order. If a child chose the "anxious" figure, a score of one was noted; if they selected the "non-anxious" figure, a score of zero was recorded. The scores from the "anxious" figure choices were totalled to determine a final score, which could range from a minimum of zero to a maximum of eight [8].

## Group interventions:

Group A (TSD): In this group, participants received a standard verbal briefing about the dental procedure through the tell-show-do technique;

Tell: The dentist explained the extraction procedure in simple terms, tailored to the child's level of understanding.

Show: Visual aids, such as dental tools (e.g., extraction forceps), were shown to the child without using them, helping to familiarize them with the instruments.

Do: The procedure was executed while ensuring to maintain a calm environment, with continuous reassurance provided to the child throughout the process.

Group B (AVD): Children in this group were provided with headphones connected mobile to watch age-appropriate cartoons or listen to soothing music during the procedure. The objective was to divert the child's attention from the dental extraction and reduce perceived anxiety by engaging them with entertaining content.

## Post-operative assessment:

Immediately after the extraction, anxiety levels were reassessed using the Venham's Picture Test. This allowed for a direct comparison of anxiety before and after the intervention within each group, as well as between the two groups.

## Statistical analysis:

Pre-operative and post-operative anxiety scores were analyzed using SPSS version 24.0. Mean scores were calculated and paired and independent samples t-tests were performed to determine statistical significance, with a p-value of less than 0.05 considered significant.

## Results:

The research involved 40 participants, consisting of 22 boys and 18 girls, evenly divided between the two groups (Table 1 - see PDF). The average ages were 6.35 for Group A and 6.51 for Group B, revealing no statistically significant difference between them according to an independent t-test (Table 2 - see PDF). On Comparison, within Group B, there was a noteworthy difference in anxiety levels before and after the operation in contrast to that no significant difference were observed within group A (Table 3 - see PDF), while the comparison between the groups indicated a statistically significant difference (Table 4 - see PDF).

## Discussion:

Dental anxiety and fear are commonly experienced by children [9]. This reaction is an emotional response to the distress related to dental treatments and procedures [10, 11]. It encompasses emotional, behavioral, physiological, and cognitive aspects that can differ among individuals. The primary role of fear and anxiety is linked to the activation of the sympathetic nervous system, causing the patient to perceive a sense of threat due to unfamiliar environments, noises in dental settings, potentially painful procedures, and past negative experiences. Additionally, pain has a psychosomatic aspect that influences how a person responds to painful stimuli, affecting their comfort levels [9]. Chhabra *et al.* in 2012 reported prevalence of dental anxiety and fear in children varies widely, with rates 5% to 33% across different countries [12]. The literature outlines several techniques for managing fear, with key concepts in behavior guidance highlighted by the American Academy of Pediatric Dentistry. Basic techniques include effective communication, the tell-show-do method, voice control, modelling, reinforcement and managing parental presence/absence. Recent behavior guidance methods consist of conscious sedation, and general anesthesia [13]. One widely used non-pharmacologic behavior management technique is the tell-show-do method, introduced by Addleston in 1959. This approach involves three key steps: first, the dentist explains the procedure to the child using age-appropriate language; second, the dentist demonstrates how the procedure will be performed; and finally, the dentist conducts the procedure exactly as it was described and shown. This technique helps alleviate anxiety by ensuring the child understands what to expect [14]. Distraction is a commonly employed technique in dental practices to help divert a child's attention away from what may be perceived as unfriendly procedures, shifting their focus to engaging and interesting distractions [15]. There are two primary types of distraction methods: audio distraction and audiovisual distraction. Audio distraction consists of music, audio presentations through headphones, and storytelling. On the other hand, AVD includes story presentations on television, virtual reality experiences, and three-dimensional video glasses [16, 17]. The present study aimed to assess and compare the effectiveness of the "Tell Show Do" technique and audiovisual distraction methods in managing anxiety levels of children undergoing primary tooth extraction. By evaluating the impact of these two approaches, the study seeks to identify which method is more effective in alleviating anxiety and enhancing the overall dental experience for young patients. The present study focused on patients aged 4 to 8 years, as children in this age group often exhibit disruptive or negative behavior, making them more challenging to manage. Radhakrishna *et al.* conducted their study within the same age range [18]. In our research, the audiovisual group showed significantly better outcomes, likely because engaging with stories, songs, or cartoons helps capture children's attention and distracts them from the anxieties associated with dental procedures. As they often close their eyes to focus, the sights and sounds of the dental treatment are effectively blocked out, further reducing anxiety. This finding is supported by research conducted by Agrawal *et al.* and Kaur *et al.* which highlighted the positive effectiveness of audiovisual distraction techniques in managing dental anxiety in children [4, 19]. The improved outcomes seen with audiovisual aids can be explained by the fact that this method engages two of the children's senses, making it easier to distract them from the anxiety associated with local anesthesia administration. Similar results were also observed in the study by Prabhakar *et al.* 2007 [1]. Liu and colleagues performed a comprehensive review assessing the effectiveness of audiovisual distraction methods for reducing dental anxiety in children. They discovered that these techniques successfully helped alleviate anxiety related to dental procedures [20]. Meanwhile, Prado *et al.* carried out a systematic review of randomized controlled trials that concentrated on distraction strategies for easing anxiety and fear in pediatric dentistry. Their results showed that a variety of distraction approaches, including both audio and visual elements, effectively diminished fear and anxiety in young patients [21]. The noted significant differences between the two groups serve as a crucial takeaway. While TSD is beneficial in providing essential information and demystifying the dental experience, AVD appears to engage children more deeply, potentially leading to better anxiety management. The data suggests that practices should consider implementing AVD as a primary strategy for anxious pediatric patients. It's essential for dental practitioners to assess each child's anxiety level and tailor the behavior management technique accordingly. While AVD may be more effective in many cases, there may still be children who respond better to TSD due to their individual personality traits and cognitive understanding. While AVD may show superior effectiveness, dental practices need to consider the accessibility of technology and the associated costs. Smaller practices might find it challenging to adopt this method if resources are limited. To build upon these findings, further research is needed to explore various factors that may influence the effectiveness of these techniques, such as age, previous dental experiences, or cultural background. Additionally, longitudinal studies could assess long-term effects on children's attitudes toward dental care.

## Conclusion:

This research work emphasizes the significance of employing effective behavior management strategies in pediatric dentistry. While both TSD and AVD have their merits, the findings support the notion that audiovisual distraction can be particularly effective in managing dental anxiety among children. By integrating these techniques into practice, dental professionals can enhance the dental experience for young patients and encourage a positive relationship with oral health care.
